# Comparison of Pollution Levels, Biomagnification Capacity, and Risk Assessments of Heavy Metals in Nearshore and Offshore Regions of the South China Sea

**DOI:** 10.3390/ijerph191912248

**Published:** 2022-09-27

**Authors:** Shaochen Yang, Kaifeng Sun, Jinling Liu, Nan Wei, Xing Zhao

**Affiliations:** 1 Hubei Key Laboratory of Critical Zone Evolution, School of Earth Sciences, China University of Geosciences, Wuhan 430074, China; 2 South China Institute of Environmental Sciences, Ministry of Ecology and Environment of the People's Republic of China, Guangzhou 510655, China; 3 Engineering Research Center of Nano-Geomaterials of Ministry of Education, China University of Geosciences, Wuhan 430074, China; 4 Key Laboratory of Functional Geomaterials in China Nonmetallic Minerals Industry, China University of Geosciences, Wuhan 430074, China; 5 College of Earth Sciences, Hebei GEO University, Shijiazhuang 050031, China

**Keywords:** heavy metals, the South China Sea, carbon stable isotope, nitrogen stable isotope, biomagnification, health risk assessments

## Abstract

Seawater and fish were collected from nearshore (Pearl River Estuarine, PRE) and offshore (middle of the South China Sea, MSCS) regions of the South China Sea (SCS) to determine the heavy metals (HMs) pollution status and biomagnification characteristics. Results show that Cu in PRE seawater was moderately contaminated. Overall pollution risk of seawater were PRE (3.32) > MSCS (0.56), whereas that of fish was MSCS (0.88) > PRE (0.42). δ^13^C and δ^15^N exhibited distinguished characteristics for PRE and MSCS fish, indicating the diverse energy sources, nitrogen sources, and food web structures of nearshore and offshore regions. Cu was biomagnified whereas Pb and Ni were biodiluted in offshore fish. Hg presented significant biomagnification in both of nearshore and offshore fish. Finally, the target hazard quotient of Hg (1.41) in MSCS fish exceeded the standard limit, which was posed by high Hg concentration and consumption rate of offshore fish.

## 1. Introduction

Heavy metals (HMs), which are distributed ubiquitously in both natural and anthropogenic regions [1,2,3], are pollutants of a global concern given their persistence, toxicity, and bioaccumulation [4]. HMs accumulate in organisms primarily through three transfer pathways: respiration, dietary ingestion, and dermal absorption [5,6]. The increase of HMs per trophic level (TL) due to dietary ingestion alone is termed biomagnification, which is the major pathway of HMs transfer within the food webs, causing high-trophic-level predators to accumulate elevated levels of HMs [7,8]. Eventually, this induces adverse effects such as cancers, deformities, and even death to high-trophic-level predators in both terrestrial and aquatic ecosystems [9,10,11].

Marine ecosystems have long and complex food web structures, which may enhance the bioaccumulation ability of HMs in marine organisms [1,12,13]. There have been reports that wild oceanic fish generally exhibited higher values of HMs such as mercury (Hg), lead (Pb), and cadmium (Cd) compared with freshwater fish [14,15,16,17]. However, the consumption of marine fish has increased over the past few decades and is considered a main protein source in many regions of the world [18]. Per capita marine fish consumption rose up from 9.9 kg in the 1960s to more than 20 kg in 2020 in the world [19]. Global marine capture fishery production peaked at 80 million tons in 2020 [19]. 

These data suggest that HMs bioaccumulation may pose unpredictable hazards to human beings with the growing consumption of seafood [1,20,21]. To better manage HM health risks and predict how HM levels in marine fish respond to future environmental change, it is essential to investigate HM pollution profiles, biomagnification, and health risks in wild marine food webs. The single factor pollution index (SFPI) and composite pollution index (CPI) were widely used for the evaluation of environmental pollution levels of a single HM and multiple HMs in different media (e.g., seawater, sediment, and organisms) [5,20]. Application of traditional stable isotopes of carbon (^13^C/^12^C = δ^13^C) is applied for identifying the dietary sources of marine organisms, and nitrogen (^15^N/^14^N = δ^15^N) is well known to determine the TL and investigate the biomagnification of contaminants for marine food webs [1,22,23,24]. Previous works determined that different HMs have distinct behaviors in the marine food web [13,25]. For instance, Liu et al. (2019) reported that Hg, copper (Cu), and Cd exhibited biomagnification, biodilution, and no biomagnification/biodilution trend, respectively, in Bohai Sea, a coastal aquatic food web of China. However, there is a lack of systemic research comparing HMs bioaccumulation across different regions (e.g., nearshore and offshore) of marine ecosystems. Differences in anthropogenic impact and diverse foraging preferences of aquatic organisms between nearshore and offshore regions could affect the HM levels or HM bioaccumulation behaviors in wild fish [13,26]. Thus, in order to understand the human exposure and risk of HMs, more research is needed into the levels, bioaccumulation, and health risks in different regions of marine food webs. 

The South China Sea (SCS) is the largest semi-enclosed sea in the western tropical Pacific Ocean around Asian developing countries, which have a crucial effect on the global ocean fishery production [27]. The Pearl River, an important terrestrial contaminant source of the SCS, has been found to input numerous HMs (e.g., Hg, Cd, Pb, etc.) into the SCS [13,28,29]. Cd, Pb, Hg, and arsenic (As) are extremely toxic to organisms, which could pose hazardous impacts to humans even with low ingestion [21]. In addition, excessive consumption of Cu and nickel (Ni) would induce toxicity to humans. Aquaculture and industrial waste around the SCS may emit elevated Cu and Ni, which raised the potential health risks of Cu and Ni [20,27]. Hence, it is significant to study Ni, Cu, As, Cd, Pb, and Hg pollution profiles in wild aquatic biota and HM bioaccumulation in food webs from nearshore and offshore regions of the SCS. To resolve this, we collected seawater and fish samples from the Pearl River Estuarine (PRE) and the middle of the SCS (MSCS) areas. We analyzed the concentrations of Ni, Cu, As, Cd, Pb, and Hg in seawater and fish, as well as C/N isotope ratios in fish samples. The aims of this study are as follows: (1) to investigate the HM profiles in fish and seawater from nearshore and offshore regions of the SCS; (2) to use C and N isotopes to differentiate diet sources and TL of marine fish between nearshore and offshore regions of the SCS; (3) to investigate the bioaccumulation of selected HMs in marine fish related to different oceanic regions of the SCS; (4) to assess the health risks of HMs in marine fish in nearshore and offshore regions of the SCS.

## 2. Materials and Methods

### 2.1. Samples Collection and Preparation

**Figure 1:**
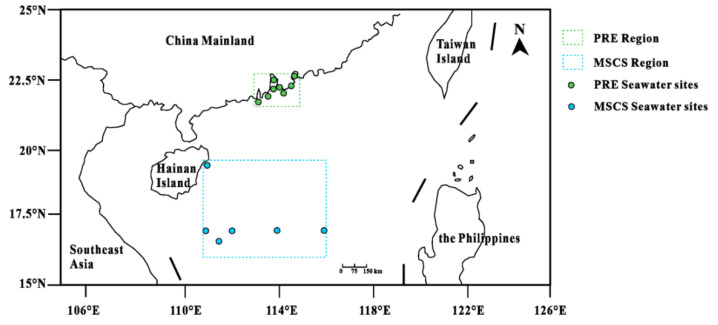
Nearshore (Pearl River Estuarine (PRE)) and offshore regions (the middle of the South China Sea, MSCS)in the South China Sea of this study. The dashed lines of green and blue represent the range of study areas. All of the fish samples were collected in the dashed line range. The green and blue spots represent the seawater sampling sites of the PRE and MSCS regions, respectively.

A total of 14 seawater samples and 84 fish samples were collected in the PRE and MSCS regions, as presented in Figure 1. Among the different sampling sites, 8 seawater samples and 28 fish samples were collected from the fishing regions of the PRE in August 2021; 6 seawater samples and 56 fish samples were collected from the fishing regions of the MSCS in June 2021. All of the fish samples collected were allowed by the marine environment survey project. Seawater samples were collected by an automatic water sampler (GHY−QCC15-10L, China). Subsequently, about 200 mL of seawater from each sampling site was acidified with 0.4 ml ultrapure HNO_3_ (65%, *v*/*v*) to immobilize the HMs, then stored in acid-washed Teflon bottles at 4 °C until chemical analysis [30]. PRE fish were collected by trawling and MSCS fish were collected by sea fishing in study areas. The basic biological parameters (e.g., species, weight, length, and moisture content, shown in Table S1) were recorded and then labeled and stored in polyethylene bags at −25 °C, awaiting chemical pretreatments [7]. In the lab, about 10 g muscle tissues of fish were separated and cleaned with deionized water. Then, the samples were measured, freeze-dried, ground, and stored at 4 °C [31]. In addition, phytoplankton samples were collected by a 200 mesh plankton net (74 mm) in two sampling sites, which were used to calculate the δ^15^N baseline value in different food webs [16].

### 2.2. Heavy Metals Analysis

The concentrations of Ni, Cu, As, Cd, and Pb in seawater and biological samples were determined by the inductively coupled plasma mass spectrometry (ICP−MS, ThermoFisher, XSeries Ⅱ, USA) facility of Faculty of Public Health, Southern Medical University. Indium and rhodium were used as the internal standards to perform the ICP-MS measurements. The detection limits of Ni, Cu, As, Cd, and Pb were 0.02, 1.47, 0.10, 0.04, and 0.05 μg/L for ICP-MS analysis, respectively. Seawater needed to be diluted to 1% ultrapure HNO_3_ solution in preparation for ICP-MS analysis [5]. Microwave digestion method was used to prepare for ICP-MS analysis of biological samples. Briefly, about 0.2−0.3 g fish tissue were weighed and placed into 50 mL Teflon digestion bottle; subsequently we added 2 mL H_2_O_2_ (30%, *v*/*v*) and 5 mL HNO_3_ (65%, *v*/*v*) under 110 °C for 1 h to remove the organic matter, then added 5 mL HNO_3_ (65%, *v*/*v*) in Teflon bottles and placed the Teflon bottles into the microwave digestion instrument. After digestion, the solution was transferred to 50 mL volumetric flasks and diluted to 50 mL with 2% HNO_3_. Hg concentration of seawater and biological samples were measured by a cold vapor atomic fluorescence spectroscopy (CVAFS, Brooks Rand Model III) facility in State Key Laboratory of Environmental Geochemistry, Institute of Geochemistry, Chinese Academy of Sciences. The detection limit of Hg was 0.01 μg/L for CVAFS method. Seawater samples were analyzed by BrCl oxidation followed by SnCl_2_ reduction, and dual amalgamation combined with CVAFS detection [28,29,32]. For biological samples’ Hg detection, about 0.2 g fish tissue were digested with 5 mL HNO_3_ (65%, *v*/*v*) at 95 °C for 3 h, and then Hg concentrations were determined by CVAFS method.

To ensure analytical quality, duplicate samples, reagent blanks, and certified reference materials (CRM, TORT 3, National Research Council Canada) were analyzed along with samples. The relative standard deviations (RSDs) of duplicate samples were lower than 10% and the reagent blanks concentration was lower than 3% of digested solution. Concentration of HMs in CRM were all between 85–112%, and are presented in Table S2. Ni, Cu, As, Cd, and Pb in seawater are exhibited as μg/L and Hg is exhibited as ng/L. Ni, Cu, As, Pb, and Hg in fish tissue are presented as mg/kg and Cd is presented as μg/kg.

### 2.3. Pollution Evaluation for Seawater and Fish

Pollution profiles in seawater and fish samples were investigated in this study. SFPI and CPI were both used for assessing the pollution level of a single HM or multiple HMs in seawater and fish samples. The SFPI and CPI were calculated by Equations (1) and (2):

(1) $$SFPI_{i}=\frac{Ci}{C_{s}}$$

(2) $$CPI=\sum_{i=1}^{n}SFPI_{i}$$

where SFPI_i_ is the SFPI index for metal i; C_i_ is the concentration of metal i in each seawater sample or the averaged concentration of metal i in each fish species; Cs is the evaluation criterion for metal i; n is the number of investigated metals in this study. The I level seawater quality standard [33] was considered as the evaluation criterion value of metals. Evaluation criteria of HMs in fish were used the reference data collected by AQSIQ (2001) and Liu et al. (2022) (Table S3) [34,35]. Relationships between contamination levels and pollution index (SFPI and CPI) are shown in Table S4. Note that the standard/risk index of As in biological samples is for inorganic As; thus, this study assumed that 5% of total As is inorganic As due to that inorganic As in seafood is generally lower than 5% [36].

### 2.4. C, N Isotope and TL Analysis for Biological Samples

Stable isotopes of C (δ^13^C) and N (δ^15^N) in biological samples were determined at the stable isotope instrument (Finnigan, MAT253, Germany) in State Key Laboratory of Environmental Geochemistry, Institute of Geochemistry, Chinese Academy of Sciences. About 0.4–0.9 mg ground fish tissue (diameter < 75 μm) were selected and packed in an aluminum foil box to prepare for the δ^13^C and δ^15^N measurements [37]. Results of δ^13^C and δ^15^N were expressed as compositions per thousand (‰) and were calculated following the previous method used by Kim et al. (2015) [37]. Moreover, δ^13^C and δ^15^N values were corrected by IAEA-certified recovery materials of C−6 (δ^13^C = −24.750‰) and N−1 (δ^15^N = 0.417‰), respectively. Variations of C and N isotopic composition in CRMs were both considered as <±0.3‰, which ensure the accuracy of δ^13^C and δ^15^N analysis.

Given that the length of food webs is normally determined as the difference between the highest and lowest TL for the considered species [38], this study estimated the TL for each biological sample by its δ^15^N values. Phytoplankton samples from the PRE and MSCS regions were collected to measure δ^15^N isotopes in this study, assuming that the phytoplankton could represent the baseline of each food web in nearshore and offshore regions, respectively [39]. The TL baseline was 1 and the δ^15^N values increased 3.4‰ per TL [24]. Thus, the TL of each sample can be calculated with Equation (3), as follows.

(3) $$TL=1+\frac{\delta^{15}N_{sample}-\delta^{15}N_{baseline}}{3.4}$$

A linear regression model, as shown in Equation (4), was used to fit the TLs and log_10_[HM_i_] concentrations for sampled species of nearshore and offshore sampling sites, where HM_i_ is the concentration of metal i in biological sample. The biomagnification factor (BF) of each HM was estimated with Equation (5), where a is the slope from the linear regression in Equation (4).

(4) $$Log_{10}[HM_{i}]=a\times TL+b$$

(5) $$BF=10^{a}$$

### 2.5. Human Health Risk Assessment

The estimated daily intake (EDI) of each HM via fish consumption was calculated using the following Equation (6) [40]:

(6) $$EDI=\frac{C_{i}\times (1-M)\times IR\times EF\times ED}{BW\times AT}$$

where C_i_ is the averaged concentration of HM i for each fish species (dw); M is the average moisture content in fish in different areas (81% and 79% for PRE and MSCS fish, respectively (Table S1)); IR is the ingestion rate for people (0.082 kg·day^−1^ for PRE region and 0.084 kg·day^−1^ for MSCS region) [41]; EF is exposure frequency (365 days/year); ED is exposure duration (70 years for adults, equivalent to the average lifetime); BW is average weight of people (70 kg in this study); and AT is average exposure time for noncarcinogens (365 days/year × ED).

In addition, the target hazard quotient (THQ) refers to the risk of noncarcinogenic effects. If THQ < 1, the exposure level is less than the reference dose [40]. This suggests that daily exposure at this level is unlikely to pose adverse effects during a person’s lifetime. It can be calculated using the following Equation (7):

(7) $$\mathrm{THQ}=\frac{\mathrm{EDI}}{\mathrm{RfD}}$$

where the EDI is the estimated daily intake; and the RfD is the reference dose [40]. Inorganic As (5% of total As) was assessed for THQ in this study [36]. The RfD values for Ni, Cu, iAs, Cd, Pb, and Hg were 0.02, 0.04, 0.0003, 0.001, 0.0015, and 0.0001 mg/kg/day, as shown in Table S3.

### 2.6. Data Analysis

Statistical significance (*p* < 0.05) of the correlation was assessed by the statistic software SPSS 19.0 (IBM, Armonk, NY, USA) for Windows system. HM concentrations in all samples were compared by the T-test method at a 5% significance level to describe statistics.

## 3. Results and Discussion

### 3.1. HM Levels and Contamination in Seawater

Table 1 lists the mean concentrations (±1 SD) of the six analyzed HMs in seawater samples of the SCS. Seawater of the PRE exhibited significantly high (*p* < 0.05) concentrations of Ni (1.35–7.98 μg/L), Cu (6.44–10.1 μg/L), As (0.503–7.60 μg/L), Cd (<LoD—1.76 μg/L), Pb (<LoD—1.24 μg/L), and Hg (1.45—10.2 ng/L) compared to MSCS seawater. This work observed Ni, Cu, and Hg in MSCS seawater ranging from 1.21–1.66 μg/L, 0.935–1.49 μg/L, and 0.770–1.78 ng/L, respectively; whereas the concentrations of As, Cd, and Pb in all of the MSCS seawater were not detected (<0.001 μg/L). Table 2 shows the results of pollution evaluation (SFPI and CPI values) of PRE and MSCS seawater. The SFPI values of six HMs in PRE seawater were Cu (1.64) > Ni (0.87) > Cd (0.27) > As (0.23) > Pb (0.20) > Hg (0.12); and in MSCS seawater were Ni (0.29) > Cu (0.25) > Hg (0.03) > As (0.00) ≈ Cd (0.00) ≈ Pb (0.00) (Table 2). CPI value of PRE seawater (CPI = 3.32) was about 6 times higher than that in MSCS seawater (CPI = 0.56).

In this study, no HM concentration in seawater exceeded the Level II of China National Environmental Quality Standards for Surface Water [33], and the CPI values in the PRE and MSCS were both at low contamination (CPI < 5), suggesting that the overall HM pollution in seawater of the SCS was at a safe level. These were consistent with the HM pollution levels in seawater of China conducted by previous works [12,30,35]. However, single pollution assessment suggested that Cu in PRE water was at moderate contamination level (1 ≤ SFPI < 3) and SFPIs values of the other five HMs in PRE seawater were also higher than those in the MSCS (Table S4). Relatively elevated HM pollution in PRE seawater could pose potential threat to the environment and should be noted in future. The rapid economic development had aggravated human activities (traffic, industrial, or agricultural) around PRE nearshore areas [35,42], which was the predominant factor causing HMs to become a main sink of anthropogenic pollutants [43]. He et al. (2021) reported that Pb and Cu in the wastewater of mariculture around the PRE area has exceeded the environmental standard limitation, which may contribute excessive Pb or Cu into PRE nearshore seawater [44]. Shi et al. (2022) also found that Pb in the PRE was mainly from anthropogenic waste input through Pb stable isotope fingerprint characteristics [45]. In addition, the watershed input could also contribute numerous HMs into PRE seawater [46,47]. According to Geng et al. (2015), the annual fluxes of Ni, Cu, Cd, and Pb were 1144, 1786, 74, and 2017 t/year, respectively [46]. A model established by Liu et al. (2021b) suggested that watershed Hg input may be an important source of coastal Hg on the global scale [47]. By contrast, MSCS was the offshore region which was distant from human activities and watershed input. Wet or dry deposition were the main pathways to input HMs into seawater [48,49]. Therefore, more elevated pollution levels of HMs in PRE seawater may be attributed to the combination of anthropogenic input and watershed contribution.

### 3.2. HM Levels and Contamination in Fish

Mean concentrations of Ni, Cu, As, Cd, Pb, and Hg in fish samples are listed in Table 1. Overall, the six studied HM concentrations exhibited considerable variation in all of fish samples. Ni, Cu, As, Cd, Pb, and Hg in PRE fish ranged from 0.0370–7.26 mg/kg, 1.33–27.4 mg/kg, 0.327–33.9 mg/kg, 0.470–59.6 μg/kg, <LoD—5.20 mg/kg, and 0.0316–0.476 mg/kg, respectively; whereas in MSCS fish they ranged from 0.203–6.26 mg/kg, 0.950–102 mg/kg, 2.75–73.4 mg/kg, <LoD—272 μg/kg, 0.265–3.493 mg/kg, and 0.0490–2.13 mg/kg, respectively. Table 2 presents the pollution evaluation results of PRE and MSCS fish. The SFPI value of six HMs in PRE fish were Ni (0.16) > Pb (0.15) > Hg (0.07) ≈ Cu (0.07) > As (0.01) > Cd (0.00), while in MSCS fish were ranked as Hg (0.39) > Cu (0.16) > Pb (0.07) > Ni (0.06) > As (0.03) > Cd (0.02). The overall CPI of PRE and MSCS fish were 0.47 and 0.73, respectively.

As shown in Table 2, SFPI of HMs in fish were lower than 1, and the CPI in fish from two regions in the SCS were lower than 5, which was consistent with fish collected in Daya Bay in previous work [35], suggesting the low contaminant level of HMs in the SCS presently. Due to aquatic organisms absorbing HMs mainly from the ambient water column [12], high HM concentrations observed in PRE seawater (as shown in Section 3.1) would expect more serious HM contamination in PRE fish. However, this study found that only Ni and Pb concentrations were significantly higher in PRE fish, compared to MSCS fish (*p* < 0.05) (Table 2). The overall pollution index in PRE fish (0.47) was also lower than that in MSCS fish (0.73). It seemed that the bioaccumulated HMs may not entirely be correlated to seawater HM levels in this work. Accumulation processes of HMs in aquatic organisms were complicated. Firstly, the bioavailability of HMs in aquatic ecosystems could affect the correlated relationship between ambient HM levels and HM bioaccumulation [50]. Meanwhile, it is widely reported that the structure of food webs in aquatic ecosystems could determine the HMs distribution in organisms [12]. Fish collected from different regions may live in different food webs and have various biomagnification ability [51], which finally affects the HM bioaccumulation. Further discussion about HMs biomagnification in different regions is given in Section 3.4. This finding suggests that at low HM pollution regions, bioaccumulated rules may have a predominant role in affecting HMs distribution in fish tissues, rather than environmental HMs exposure.

### 3.3. Geographical Differences of δ^13^C, δ^15^N, and TL

**Figure 2:**
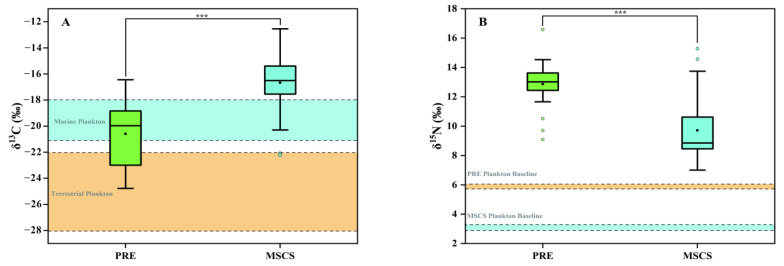
(**A**) Box plot of δ^13^C values in fish collected from the Pearl River Estuarine (PRE) and the middle of the South China Sea (MSCS). The cyan and brown areas represent the range of δ^13^C values in marine plankton and terrestrial plankton reported by previous works [52,53]. (**B**) Box plot of δ^15^N values in fish collected from the PRE and the MSCS. The dashed lines of cyan and brown represent the baseline δ^15^N in phytoplankton collected from the MSCS and the PRE, respectively.*** represents that the data are significantly different between the PRE and the MSCS (*p* < 0.001).

δ^13^C, δ^15^N values and TL for fish collected in the PRE and MSCS are given in Figure 2. The range of δ^13^C values in PRE fish were from −24.77‰ to −16.44‰; whereas in MSCS fish, they ranged from −22.24‰ to −12.54‰. Significant differences (*p* < 0.05) of δ^13^C values were found between PRE and MSCS fish, which were MSCS (−16.67‰ ± 2.00‰) > PRE (−20.59‰ ± 2.43‰) (Figure 2A).

Due to less enrichment or depletion of δ^13^C during trophic transfer, δ^13^C values are an efficient tool to track diet sources of aquatic organisms [23]. A wide range of values of δ^13^C in marine fish were widely reported in the Yellow Sea (−27.62 ‰ to −14.54 ‰) [54], East China Sea (−20.87‰ to −15.04‰) [1], South China Sea (−21.30‰ to −19.08‰) [55], and even deep waters of Sulu Sea and the Celebes Sea (−19.30‰ to −14.37‰) [56]. By contrast, wild fish collected in freshwater ecosystems, such as Poyang Lake (−29.4‰ to −24.5‰) [57] and Taihu Lake (−25.7‰ to −18.1‰) [58], generally exhibited relatively lower values of δ^13^C. The variances observed in the stable isotope ratio of δ^13^C may predominately reflect significant differences of primary organic matter in the daily diets of fish from different geographical regions [40]. It has been reported that different primary organic matters, e.g., marine plankton (−21‰ to −18‰), river plankton (−28‰ to −22‰), and terrestrial plants (−27‰ to −25‰), have distinct δ^13^C values [52,53]. Hence, significantly different δ^13^C values in PRE and MSCS fish may be attributed to the distinct carbon sources in nearshore and offshore areas. PRE fish possessed obviously negative δ^13^C values compared with MSCS fish, which suggested that more terrestrial organic matter was assimilated by fish living in nearshore regions, while MSCS fish displayed the relatively positive δ^13^C values, indicating the diet sources of marine organic carbon [56].

The δ^15^N values of PRE fish (from 9.10‰ to 16.58‰; 12.87‰ ± 1.47‰) were significantly higher (*p* < 0.05) than those in MSCS fish (from 7.01‰ to 15.28‰; 9.71‰ ± 2.06‰), as shown in (Figure 2B). The δ^15^N values in the SCS fish collected in this study were generally in the range of those previously reported in Chinese coastal fish (from 8.00‰ to 15.62‰) [1,59], except for one *Scoliodon laticaudus* sample in the PRE that exhibited δ^15^N value of 16.6‰. Both δ^15^N enrichment during trophic transfer and varied δ^15^N baseline values in the primary producer (mediated by varied primary nitrogen sources) are able to alter the δ^15^N values in aquatic food webs [23]. This work observed that the δ^15^N baseline in the PRE (δ^15^N = 5.72) was about two times higher than that in the MSCS (δ^15^N = 2.69) (Figure 2). Human N sources, such as organic matter in the wastewater, have been reported to contain heavier N isotope, whereas N in precipitation has lighter N isotope [60]. Different N baseline in the PRE and MSCS may be attributed to primary nitrogenous materials in nearshore regions that were certainly contributed by anthropogenic activities; by contrast, offshore regions were generally from natural wet deposition [61]. Given that the TL of phytoplankton was generally considered as 1 (Section 2.4), the TL in 9 PRE fish species varied between 2.86 and 3.59 (3.17 ± 0.27), which was comparable to 25 fish species in the MSCS (from 2.56 to 4.46; 3.22 ± 0.55), suggesting that the enrichment of δ^15^N in nearshore and offshore food webs was similar. Thus, it could be concluded that anthropogenic activities have impact on δ^15^N baseline values in nearshore areas of the SCS, which eventually lead to high δ^15^N values in PRE fish. We conclude that the combination of C and N stable isotopes is a powerful tool for determining the geographical origins and living habitats of wild fish from nearshore and offshore marine regions.

### 3.4. Biomagnification, Biodilution, and Correlations of HMs

Table 3 shows the regression analysis results between logarithm of HM contents and TLs. The BF greater than 1 indicates that HMs are able to magnify/accumulate in food webs, while the BF less than 1 suggests the dilution/elimination of the HM from the food webs [12]. This work observed that Hg (BF = 2.14; *p* < 0.05) in PRE fish, as well as Cu (BF = 2.69; *p* < 0.01) and Hg (BF = 2.51; *p* < 0.01) in MSCS fish, displayed significant biomagnification; while Ni (BF = 0.56; *p* < 0.01) and Pb (BF = 0.69; *p* < 0.01) of MSCS fish displayed an obvious decrease with TL (Table 3). Neither As nor Cd showed biomagnification/biodilution along the food chains or in the entire food web of the SCS. Hg exhibited biomagnification in both the PRE and the MSCS, which was consistent with previous works conducted in freshwater lakes, coastal areas, open ocean, and even Arctic areas [12,62,63]. This was attributed to the fact that most of Hg is methylmercury (MeHg), and MeHg has the potent ability to bond to thiol in organism tissues and biomagnify along food webs [13]. This work observed that BF in the MSCS (2.51) was slightly higher than in the PRE (2.14), which may explain the relatively high Hg levels in offshore fish. The variances of BF between PRE and MSCS may be affected by physicochemical and biological characteristics of the ecosystem and human activities impact [51]. Further works need to investigate the factors controlling the BF in the SCS to reduce Hg levels in aquatic organisms. By contrast, the TL-dependent biomagnification or biodilution of Cu, Ni, and Pb exhibited different behaviors between PRE and MSCS fish. Influenced by the divergence of feeding habit, dissimilar transfer behaviors of HMs at varied TLs were exhibited [64]. Biodilution of Ni, Cu, and Pb in top predators were widely reported by previous works [65,66,67], which may be attributed to fish generally having effective metabolic mechanisms to regulate the Pb and Ni concentrations in the body. These HMs, however, exhibited biomagnification in phytoplankton, zooplankton, and crustaceans [67]. Thus, additional studies could pay attention to investigating the predominant factors affecting the bioaccumulation of Cu, Ni, and Pb among different organism species.

**Figure 3:**
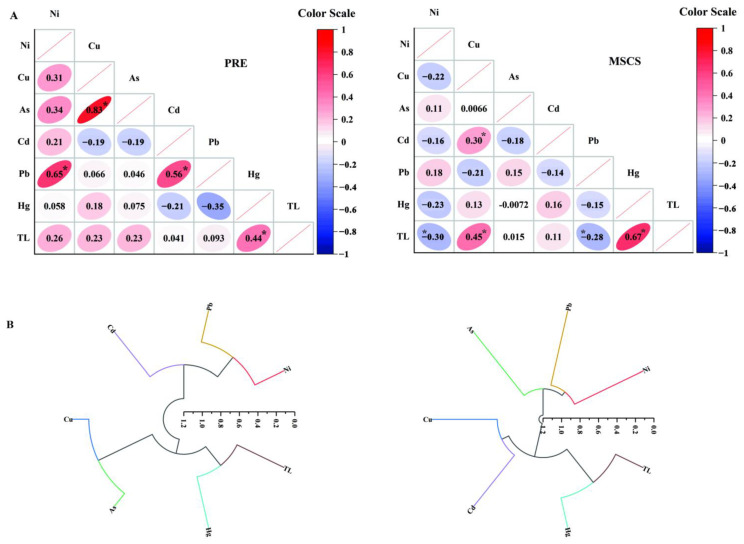
(**A**) Correlation analysis of the heavy metals (HMs) and trophic level (TL) in fish from the Pearl River Estuarine (PRE) and the middle of the South China Sea (MSCS). Data in the ellipses represent the correlation coefficient between the two variables. * Means the significance value *p* < 0.05. (**B**) Cluster analysis of the HMs and TL in fish from the PRE and the MSCS.

This study also presents the results of correlation analysis (CA) and hierarchical clustering (HC) of HMs and TL of fish in Figure 3. CA results among HMs and TL indicate that complex correlations exist among those variables in fish samples (Figure 3A). The relationship of HMs and TL were similar to the biomagnification model in Table 3. In addition, Ni–Pb, Cu–As, and Cd–Pb in PRE fish, as well as Cu–Cd, showed significant positive correlations (*p* < 0.05). Positive correlations in environmental media generally represent similar sources of HMs [44]. However, according to Section 3.2, HMs bioaccumulated in marine fish may have experienced more complicated processes. All the biology, ecology, and physiology of the organisms could play key roles in HM bioaccumulation [51]. Thus, the correlation of HMs may suggest the similar bioaccumulate processes of those organisms, rather than similar sources. For instance, positive Cu–Cd correlation could be explained by the efficient uptake mechanisms for Cu that cannot discriminate between Cu and Cd in some aquatic species [68]; and positive Ni–Pb correlation may be attributed to the similar elimination efficiency of Ni and Pb in fish [67]. HC analysis was performed on the dataset of HM concentrations (Figure 3B). It should be noted that Hg is the only HM which clustered with TL in the PRE and MSCS regions. This was consistent with the biomagnification and correlation analysis results (Table 3). Overall, we conclude that, compared to other HMs, the potent biomagnification ability of Hg along with TL needs to be investigated, regardless of nearshore or offshore regions of marine ecosystems.

### 3.5. HMs Health Risks of Marine Fish

**Figure 4:**
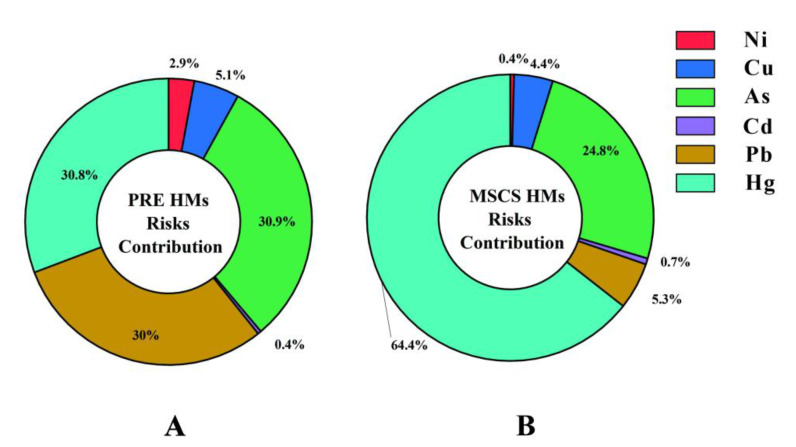
Comparison of target hazard quotient (THQ) and relative contribution of 6 analyzed heavy metals (HMs) in the Pearl River Estuarine (PRE, (**A**)) and the middle of the South China Sea (MSCS, (**B**)) fish.

The EDI and THQ for fish collected from the PRE and MSCS regions could evaluate the HM risks of nearshore and offshore fish intake, which are listed in Table 2 and Figure 4. Results of EDI showed that Cu had the highest daily intake, which were 0.016 and 0.038 mg/day/kg in nearshore and offshore areas, respectively. THQ of HMs in PRE fish were As (0.25) ≈ Hg (0.25) > Pb (0.24) > Cu (0.04) > Ni (0.02) > Cd (0.00); while in MSCS fish were Hg (1.41) > As (0.54) > Pb (0.11) > Cu (0.10) > Cd (0.02) > Ni (0.01). The ΣTHQ of six HMs in PRE and MSCS fish were 0.80 and 2.19, respectively (Table 2). It has been reported that HMs health risks of coastal fish in China were generally at safe level [21,30,54,69]. The THQ of all HMs in fish collected in the PRE were less than 1, which were similar to fish collected in the Yellow River Estuarine [54], East China Sea [21], and Yellow Sea [30] of China, indicating no significant risk of HMs from consuming marine fish from the PRE (Figure 4). Similarly, potential health risks of Ni, Cu, As, Cd, and Pb in MSCS fish were at safe levels (THQ < 1) [35,40]. It should be noted that the THQ of Hg (1.41) in MSCS fish has exceeded the safe limit (Table 3). Hg is easy to biomagnify in marine food webs and high levels of Hg are generally determined in fish with slow growth rate [17,51]. Low nutrient levels in the MSCS may reduce the growth rate of fish, which elevates Hg levels and health risks in MSCS fish [13]. Chronic Hg exposure could impact the liver and pituitary gland, which lead to a compromise of the immune and neuro system [70]. This study therefore proposes that there are potential risks of developing chronic systemic effects due to Hg intake from SCS offshore fish. In addition, we found that the health risks of Hg in large (body length > 10 cm; THQ = 1.41) and small (body length < 10 cm; THQ = 1.43) fish are similar in MSCS regions, which could be due to growth dilution reducing Hg concentration in larger fish [51], suggesting that health risks for both large and small fish need to be addressed, although Gu et al. (2015) determined no obvious health risks from the intake of HMs through offshore SCS fish consumption, which was not consistent with this study. This may be attributed to the fact that only Ni, Cu, Cd, Mn, Pb, and Cr were detected by Gu et al., who did not assess the risks of Hg [69]. According to Section 3.2, the HM pollution levels in MSCS fish have not exceeded the limit; however, the personal seafood daily intake in Hainan province is over 20-folds higher than that in Chinese inland areas (3 g/day) [71,72]. Hence, we determined that extremely high daily intake of seafood may pose potential toxicity to local people living in Hainan, and this needs to be paid more attention.

## 4. Conclusions

This study demonstrates that the pollution profiles of HMs in seawater of the PRE and the MSCS are at a safe level, except for the moderate pollution of Cu in PRE seawater. The CPI values indicate that fish in the MSCS, rather than the PRE, bioaccumulate more HMs. Distinct C and N stable isotope ratios reveal the differences of habitat in marine fish from nearshore and offshore regions of the SCS. Compared to other HMs, Hg is the unique HM which exhibits biomagnification in both nearshore and offshore areas and is correlated with TL. Environmental behaviors of Hg in marine ecosystems need to be investigated in future. In addition, the THQ of Hg in offshore (THQ of Hg = 1.41) fish of the SCS have exceeded the safe limit. Given the significant biomagnification ability and potential health risks of Hg, we propose that further works need to pay more attention to tracing bioaccumulated Hg sources and developing consumption advisories of marine fish, to minimize the potential environmental impacts of Hg.

## Figures and Tables

**Table 1:** Heavy metal concentrations (mean ± 1 SD) in seawater and each fish species (dry weight) collected from the Pearl River Estuarine (PRE) and middle of the South China Sea (MSCS).

	*n*	Ni	Cu	As	Cd	Pb	Hg
Seawater							
PRE	8	4.36 ± 2.37	8.18 ± 1.42	4.50 ± 2.39	0.27 ± 0.61	0.20 ± 0.43	5.81 ± 3.32
MSCS	6	1.45 ± 0.12	1.24 ± 0.23	N.D.	N.D.	N.D.	1.27 ± 0.39
Fish							
*Scoliodon laticaudus*	6	2.30 ± 1.95	7.55 ± 5.30	6.16 ± 3.29	7.35 ± 16.60	0.94 ± 1.23	0.37 ± 0.17
*Coilia mystus*	4	3.07 ± 2.85	4.15 ± 1.91	3.44 ± 0.78	18.43 ± 21.25	2.93 ± 2.26	0.04 ± 0.08
*Konosirus punctatus*	2	0.83 ± 0.22	4.15 ± 1.96	5.56 ± 0.46	30.13 ± 41.64	1.56 ± 1.41	0.04 ± 0.02
*Thryssa kammalensis*	2	0.80 ± 0.29	6.09 ± 3.23	3.16 ± 0.00	2.00 ± 1.75	2.17 ± 0.68	0.07 ± 0.00
*Pennahia argentata*	3	0.73 ± 0.58	6.70 ± 3.43	2.77 ± 2.30	1.68 ± 1.80	0.14 ± 0.20	0.13 ± 0.12
*Sardina*	7	0.53 ± 0.37	3.07 ± 1.51	2.36 ± 1.40	7.80 ± 13.33	0.39 ± 0.59	0.09 ± 0.05
*Harpadon nehereus*	1	6.21	27.36	33.91	1.02	2.55	0.10
*Collichthys niveatus*	1	0.71	5.20	1.57	58.15	1.94	0.05
*Acanthopagrus schlegelii*	2	3.46 ± 1.81	2.11 ± 0.47	0.90 ± 0.81	1.14 ± 0.78	1.91 ± 2.70	0.10 ± 0.08
Total of the PRE	28	1.75 ± 1.93	5.74 ± 5.39	4.65 ± 6.23	10.83 ± 18.84	1.30 ± 1.50	0.14 ± 0.13
*Caranx ignobilis*	3	0.31 ± 0.04	24.10 ± 5.64	3.59 ± 0.62	13.09 ± 12.43	0.49 ± 0.13	0.59 ± 0.33
*Acanthopagrus schlegelii*	2	0.44 ± 0.10	20.41 ± 0.94	5.94 ± 0.41	163.51 ± 153.88	0.49 ± 0.13	0.59 ± 0.33
*Pampus chinensis*	2	0.38 ± 0.09	8.12 ± 2.74	7.43 ± 0.09	2.39 ± 1.90	0.45 ± 0.07	0.27 ± 0.00
*Pennahia argentata*	2	0.48 ± 0.01	83.50 ± 25.64	15.85 ± 0.27	120.35 ± 55.51	0.47 ± 0.00	0.31 ± 0.06
*Pomadasys maculatus*	1	0.64	48.58	17.47	0.74	0.76	0.236
*Trichiurus lepturus*	3	0.26 ± 0.05	14.98 ± 15.18	3.60 ± 0.57	161.12 ± 51.56	0.36 ± 0.07	1.37 ± 0.18
*Pentapus*	5	0.77 ± 0.25	5.24 ± 2.60	14.08 ± 5.78	2.57 ± 4.05	0.97 ± 0.40	0.25 ± 0.13
*Cephalopholis*	4	2.33 ± 2.62	2.32 ± 0.84	13.45 ± 4.31	32.04 ± 54.40	1.44 ± 1.38	0.27 ± 0.17
*Priacanthus*	2	0.78 ± 0.40	4.91 ± 1.96	4.36 ± 0.26	2.57 ± 4.05	0.97 ± 0.40	0.25 ± 0.13
*Neoniphon*	1	1.15	15.22	16.68	1.66	0.99	1.05
*Epinephelus*	5	0.86 ± 0.32	6.58 ± 5.02	8.35 ± 3.58	25.63 ± 39.51	0.66 ± 0.25	0.57 ± 0.16
*Balistapus*	2	0.44 ± 0.12	12.23 ± 5.44	45.39 ± 39.66	9.65 ± 12.66	0.94 ± 0.20	0.29 ± 0.13
*Sebastiscus*	3	0.98 ± 0.20	4.23 ± 2.33	9.23 ± 5.35	7.20 ± 5.31	0.68 ± 0.03	0.21 ± 0.13
*Decapterus maruadsi*	2	0.34 ± 0.07	17.74 ± 10.63	4.03 ± 0.86	20.31 ± 8.79	0.35 ± 0.05	0.23 ± 0.04
*Thunnini*	2	0.53 ± 0.18	11.31 ± 0.13	8.03 ± 0.13	69.38 ± 28.37	0.58 ± 0.22	1.49 ± 0.44
*Ilisha elongata Bennett*	2	0.56 ± 0.02	20.70 ± 0.55	2.78 ± 0.04	3.26 ± 3.46	0.60 ± 0.15	0.20 ± 0.01
*Scomberomorus niphonius*	3	0.33 ± 0.10	28.74 ± 9.62	20.20 ± 27.28	16.24 ± 17.45	0.46 ± 0.28	0.92 ± 0.75
*Apogonidae*	2	1.12 ± 0.30	7.79 ± 7.98	13.35 ± 7.25	22.40 ± 30.67	1.32 ± 0.97	0.80 ± 0.36
*Myripristis berndti*	1	0.74	1.89	29.04	0.85	0.75	2.13
*Hemibarbus*	2	1.32 ± 0.60	3.41 ± 0.67	13.66 ± 9.65	5.66 ± 0.41	0.61 ± 0.19	0.36 ± 0.11
*Lethrinus haematopterus*	3	0.82 ± 0.24	2.83 ± 2.29	8.88 ± 3.03	13.00 ± 21.01	0.67 ± 0.13	0.45 ± 0.10
*Variola*	1	0.78	11.52	12.16	0.78	1.20	0.20
*Upeneus*	1	0.94	4.40	22.50	14.30	0.55	0.27
*Mullidae*	1	0.40	14.16	10.66	0.61	0.52	0.47
*Ariussinensis Lacepede*	1	0.49	6.13	13.00	0.53	0.40	0.41
Total of MSCS	56	0.78 ± 0.82	13.8 ± 17.3	11.9 ± 11.7	31.3 ± 56.9	0.71 ± 0.490	0.570 ± 0.468

Note: Ni, Cu, As, Cd, and Pb in seawater are exhibited as μg/L and Hg is exhibited as ng/L. Ni, Cu, As, Pb, and Hg in fish tissue are presented as mg/kg and Cd is presented as μg/kg.

**Table 2:** Results of single factor pollution index (SFPI) and composite pollution index (CPI) in seawater as well as SFPI, CPI, estimated daily intake (EDI), and target hazard quotient (THQ) in fish collected from the Pearl River Estuarine (PRE) and the middle of the South China Sea (MSCS).

	Ni	Cu	As	Cd	Pb	Hg	CPI/ΣTHQ
SFPI_s_							
PRE	0.87	1.64	0.23	0.27	0.20	0.12	3.32
MSCS	0.29	0.25	0.00	0.00	0.00	0.03	0.56
SFPI_f_							
PRE	0.16	0.07	0.01	0.00	0.15	0.07	0.47
MSCS	0.06	0.16	0.03	0.02	0.07	0.39	0.73
EDI							
PRE	4.61 × 10^−4^	1.64 × 10^−3^	1.48× 10^−3^	3.16 × 10^−6^	3.59 × 10^−4^	2.46 × 10^−5^	-
MSCS	1.83 × 10^−4^	3.84 × 10^−3^	3.26 × 10^−3^	1.50 × 10^−5^	1.74 × 10^−4^	1.41 × 10^−4^	-
THQ							
PRE	2.31 × 10^−2^	4.10 × 10^−2^	2.47 × 10^−1^	3.16 × 10^−3^	2.40 × 10^−1^	2.46 × 10^−1^	1.05 × 10^0^
MSCS	9.17 × 10^−3^	9.60 × 10^−2^	5.44 × 10^−1^	1.50 × 10^−2^	1.16 × 10^−1^	1.44 × 10^0^	2.74 × 10^0^

Note: SFPI_s_ means the SFPI of seawater. SFPI_f_ means the SFPI of fish. The EDI and THQ in this table are expressed as scientific notation. The unit of EDI is mg/day/kg. Other indexes have no unit.

**Table 3:** Biomagnification factor (BF) and regression analysis (slope, intercept, correlation coefficient (R), *p*-value of slope) between logarithm of heavy metal concentrations and trophic levels for fish collected in Pearl River Estuarine (PRE) and the middle of the South China Sea (MSCS).

	Ni	Cu	As	Cd	Pb	Hg
PRE						
Slope	0.25	0.11	0.07	−0.16	−0.27	0.33
Intercept	−0.75	0.29	0.25	−2.18	0.18	−2.02
BF	1.78	1.29	1.17	0.69	0.54	2.14
R	0.23	0.15	0.07	0.09	0.09	0.39
*p*	0.25	0.44	0.74	0.65	0.64	0.04 *
MSCS						
Slope	−0.25	0.43	−0.08	0.29	−0.16	0.40
Intercept	0.57	−0.41	1.19	−3.16	0.28	−1.61
BF	0.56	2.69	0.83	1.95	0.69	2.51
R	0.53	0.52	0.14	0.18	0.43	0.66
*p*	<0.01 *	<0.01 *	0.32	0.18	<0.01 *	<0.01 *

Note: The slope and intercept represent the *a* and *b* in Equation (4). * Means the result is statistically significant.

## Supplementary Materials

The following supporting information can be downloaded at: https://www.mdpi.com/article/10.3390/ijerph191912248/s1, Table S1: Detailed information of species, length (cm), weight (g), and moisture content (%) in fish captured from Pearl River Estuarine (PRE) and the middle of the South China Sea (MSCS); Table S2: Standard value and recovery (%) of 6 heavy metals (HMs) in certified reference material (TORT 3) in this study; Table S3: Reference standard for pollution status and ecological risk assessment of heavy metals in seawater and fish (wet weight) in this study; Table S4: Relationship between evaluations and pollution levels.
