# Supplementary material for: Comparison of Pollution Levels, Biomagnification Capacity, and Risk Assessments of Heavy Metals in Nearshore and Offshore Regions of the South China Sea
Source: Int J Environ Res Public Health. 2022 Sep 27;19(19):12248. doi: 10.3390/ijerph191912248 (PMC9565928; doi:10.3390/ijerph191912248)
Supplement: Supplementary file 1 [file ijerph-19-12248-s001.zip › ijerph-1916319-supplementary.pdf]

Supporting information for: Comparison of pollution levels, biomagnification capacity, and risk assessments of heavy metals in nearshore and offshore regions of the South China Sea

Shaochen Yang[1,2,3,4], Kaifeng Sun[2*], Jinling Liu[1,3,4], Nan Wei[2], Xing Zhao[2,5]

1. Hubei Key Laboratory of Critical Zone Evolution, School of Earth Sciences, China University of Geosciences, Wuhan, 430074, China

2. South China Institute of Environmental Sciences, MEP, Guangzhou 510655, China

3. Engineering Research Center of Nano-Geomaterials of Ministry of Education, China University of Geosciences, Wuhan 430074, China

4. Key Laboratory of Functional Geomaterials in China Nonmetallic Minerals Industry, China University of Geosciences, Wuhan 430074, China

5. College of Earth Sciences，Hebei GEO University，Shijiazhuang，Hebei，050031，China

*Author for correspondence. Tel: 86-020-29119769; Email: sunkaifeng@scies.org

This supporting information including 6 pages, 4 tables.

Table S1. Detailed information of species, length (cm), weight (g) and moisture content (%) in fish captured from Pearl River Estuarine (PRE) and the Middle of South China Sea (MSCS)

Samples No.	Species	Length (cm)	Weight (g)	Moisture (%)
PRE1	*Scoliodon laticaudus*	51	590	85%
PRE2	*Scoliodon laticaudus*	48	443	86%
PRE3	*Scoliodon laticaudus*	42	389	78%
PRE4	*Scoliodon laticaudus*	40	410	77%
PRE5	*Scoliodon laticaudus*	35	215	77%
PRE6	*Scoliodon laticaudus*	43	442	81%
PRE7	*Coilia mystus*	7	41	86%
PRE8	*Coilia mystus*	8	47	75%
PRE9	*Coilia mystus*	9	41	84%
PRE10	*Coilia mystus*	9	66	76%
PRE11	*Thryssa kammalensis*	10	52	83%
PRE12	*Thryssa kammalensis*	9	67	84%
PRE13	*Konosirus punctatus*	12	144	84%
PRE14	*Konosirus punctatus*	6	35	86%
PRE15	*Pennahia argentata*	10	116	79%
PRE16	*Pennahia argentata*	9	86	80%
PRE17	*Pennahia argentata*	7	58	84%
PRE18	*Sardina*	10	81	75%
PRE19	*Sardina*	10	87	86%
PRE20	*Sardina*	9	67	76%
PRE21	*Sardina*	9	47	86%
PRE22	*Sardina*	6	33	81%
PRE23	*Sardina*	8	71	83%
PRE24	*Sardina*	9	68	86%
PRE25	*Harpadon nehereus*	13	56	77%
PRE26	*Collichthys niveatus*	8	52	76%
PRE27	*Acanthopagrus schlegelii*	12	61	79%
PRE28	*Acanthopagrus schlegelii*	8	71	76%
MSCS1	*Decapterus maruadsi*	28	125	81%
MSCS2	*Decapterus maruadsi*	30	153	74%
MSCS3	*Thunnini*	85	677	75%
MSCS4	*Thunnini*	93	812	86%
MSCS5	*Ilisha elongataBennett*	38	370	79%
MSCS6	*Ilisha elongataBennett*	36	311	79%
MSCS7	*Scomberomorus niphonius*	29	415	72%
MSCS8	*Scomberomorus niphonius*	23	304	85%
MSCS9	*Scomberomorus niphonius*	24	306	83%

Samples No.	Species	Length (cm)	Weight (g)	Moisture (%)
MSCS10	*Pennahia argentata*	19	210	81%
MSCS11	*Pennahia argentata*	18	170	85%
MSCS12	*Pampus chinensis*	23	125	77%
MSCS13	*Pampus chinensis*	23	144	80%
MSCS14	*Caranx ignobilis*	22	164	75%
MSCS15	*Caranx ignobilis*	23	166	75%
MSCS16	*Caranx ignobilis*	21	124	78%
MSCS17	*Acanthopagrus schlegelii*	19	198	79%
MSCS18	*Acanthopagrus schlegelii*	19	205	74%
MSCS19	*Pomadasys maculatus*	15	244	82%
MSCS20	*Trichiurus lepturus*	82	450	83%
MSCS21	*Trichiurus lepturus*	68	355	74%
MSCS22	*Trichiurus lepturus*	79	416	72%
MSCS23	*Pentapus*	10	68	88%
MSCS24	*Pentapus*	8	55	79%
MSCS25	*Pentapuss*	11	85	82%
MSCS26	*Pentapus*	10	57	79%
MSCS27	*Pentapus*	8	55	80%
MSCS28	*Cephalopholis*	10	48	78%
MSCS29	*Priacanthus*	9	46	71%
MSCS30	*Cephalopholis*	10	64	82%
MSCS31	*Cephalopholis*	9	57	77%
MSCS32	*Neoniphon*	14	145	77%
MSCS33	*Apogonidae*	8	27	80%
MSCS34	*Myripristis berndti*	9	85	82%
MSCS35	*Apogonidae*	16	131	76%
MSCS36	*Epinephelus*	9	75	77%
MSCS37	*Epinephelus*	9	62	86%
MSCS38	*Epinephelus*	10	79	77%
MSCS39	*Hemibarbus*	17	141	78%
MSCS40	*Variola*	9	52	72%
MSCS41	*Lethrinus haematopterus*	17	153	82%
MSCS42	*Lethrinus haematopterus*	17	138	82%
MSCS43	*Lethrinus haematopterus*	15	115	79%
MSCS44	*Sebastiscus*	11	97	78%
MSCS45	*Sebastiscus*	9	45	87%
MSCS46	*Sebastiscus*	8	41	83%
MSCS47	*Ariussinensis Lacepede*	17	149	79%
MSCS48	*Priacanthus*	9	57	73%
MSCS49	*Balistapus*	16	158	81%
MSCS50	*Balistapus*	15	145	79%
MSCS51	*Mullidae*	19	183	71%
MSCS52	*Upeneus*	19	131	78%

Samples No.	Species	Length (cm)	Weight (g)	Moisture (%)
MSCS53	*Epinephelus*	7	40	80%
MSCS54	*Epinephelus*	8	44	79%
MSCS55	*Hemibarbus*	10	54	79%
MSCS56	*Cephalopholis*	8	31	78%

Table S2. Standard value and recovery (%) of 6 heavy metals (HMs) in certified reference material (TORT 3) in this study.

HMs	Unit	Standard value	Measured value	Recovery
Ni	mg/kg	5.30	5.07	95.67%
Cu	mg/kg	497	426.25	85.76%
As	mg/kg	54.9	54.94	100.08%
Se	mg/kg	10.9	12.12	111.18%
Cd	mg/kg	42.3	37.41	88.43%
Pb	mg/kg	0.230	0.24	108.88%
Hg	mg/kg	0.292	0.260	89.04%

Table S3 Reference standard for pollution status and ecological risk assessment of heavy metals in seawater and fish (wet weight) in this study.

	Ni	Cu	As (IAs)	Cd	Pb	Hg
Seawater quality standard (μg/L)	5.0	5.0	20	1.0	1.0	0.050
Fish quality standard (mg/kg)	2.5	20	5.0	0.60	2.0	0.30
Rfd (mg/kg/day)	0.02	0.04	0.0003	0.001	0.0015	0.0001

Note: IAs means the inorganic As. Rfd means the reference dose.

Table S4 Relationship between evaluations and pollution levels.

Index	Value	Contaminant level
SFPI	<1	Low
	1≤SFPI<3	Moderate
	3≤SFPI<6	Considerable
	SFPI≥6	Very high
CPI	CPI<5	Low
	5≤CPI<10	Moderate
	10≤CPI<20	Considerable
	CPI≥20	Very high
THQ	<1	no risk
	1≤THQ<10	low risk
	10≤THQ<20	moderate risk
	>20	high risk

Note: The SFPI, CPI and THQ represent the single factor pollution index, composite pollution index and target hazard quotient, respectively.
